# Supplementary material for: Effects of Rearing Aviary Style and Genetic Strain on the Locomotion and Musculoskeletal Characteristics of Layer Pullets
Source: Animals (Basel). 2021 Feb 27;11(3):634. doi: 10.3390/ani11030634 (PMC7997280; doi:10.3390/ani11030634)
Supplement: Supplementary file 1 [file animals-11-00634-s001.zip › APufall_SupplementaryMaterial_S1.docx]

| **Flock** | | **Style** | **Genetic Line** | **Colour** | **Brooder Compartments Opened (days)** | **Aisle Width (m)** | **Aisle Length (m)** | **Total Chicks Placed** | **Average Group Size** | **Natural Light** | **Migration Fences** | **Visits When Ramps Available** |
| --- | --- | --- | --- | --- | --- | --- | --- | --- | --- | --- | --- | --- |
| 1A | 1 | | Lohmann | Brown | 46 | 1.6 | 102.4 | 29 600 | 14 800 | no | yes | 2, 3 |
| 1B | 1 | | Dekalb | White | 48 | 1.5 | 122.8 | 32 742 | 10 914 | no | yes | 2,3 |
| 1C^1^ | 1 | | Lohmann | Brown | 33 | 1.9 | 104.0 | 47 000 | 2 500 | no | yes | 2 |
| 1D^1^ | 1 | | Lohmann | White | 33 | 1.9 | 104.0 | 47 000 | 3 000 | no | yes | 2 |
| 1E^1^ | 1 | | Dekalb | White | 28 | 2.3 | 104.0 | 47 000 | 5 167 | no | yes | 2 |
|  |  | |  |  |  |  |  |  |  |  |  |  |
| 2A | 2 | | Lohmann | White | 28 | 1.4 | 50.1 | 43 000 | 5 591 | no | no | 2, 3 |
| 2B | 2 | | Bovan | Brown | 28 | 1.7 | 64.5 | 35 506 | 6 642 | no | no | 2, 3 |
| 2C^2^ | 2 | | Lohmann | White | 28 | 21.3 | 82.7 | 39 200 | 7 350 | yes | no | 2, 3 |
| 2D | 2 | | Shaver | White | 22 | 1.4 | 33.6 | 11 200 | 4 200 | no | no | none |
| 2E^3^ | 2 | | Lohmann | Brown | 28 | 2.0 | 57.9 | 22 470 | 4 214 | yes | no | 2,3 |
|  |  | |  |  |  |  |  |  |  |  |  |  |
| 3A | 3 | | Lohmann | White | 42 | 2.7 | 48.0 | 14 240 | 14 240 | no | no | 1,2 |
| 3B^3^ | 3 | | Lohmann | Brown | 49 | 2.0 | 67.8 | 20 700 | 20 700 | yes | no | 2 |
| 3C^4^ | 3 | | Lohmann | Brown | 91 | 2.7 | 42.7 | 9 200 | 9 200 | no | no | 2 |
| 3D | 3 | | ISA | Brown | 45 | 3.3 | 7.2 | 700 | 700 | no | no | 2,3 |
| 3E | 3 | | Lohmann | White | 46 | 1.8 | 34.0 | 11 730 | 11 730 | no | no | 1,2,3 |
| ^1^Flocks 1C, 1D, and 1E were simultaneously housed same building and separated by migration fences. There were 10 000 Lohmann Brown, 6 000 Lohmann White, and 31 000 Dekalb White chicks placed.  ^2^Non-organic flock provided natural light  ^3^Organic flocks provided natural light  ^4^Organic flock not provided natural light | | | | | | | | | | | | |

**Table S1.** General management for all flocks
