# Supplementary material for: Effects of Rearing Aviary Style and Genetic Strain on the Locomotion and Musculoskeletal Characteristics of Layer Pullets
Source: Animals (Basel). 2021 Feb 27;11(3):634. doi: 10.3390/ani11030634 (PMC7997280; doi:10.3390/ani11030634)
Supplement: Supplementary file 1 [file animals-11-00634-s001.zip › APufall_SupplementaryMaterial_S1.pdf]

Table S1. General management for all flocks

Flock	Style	Genetic Line	Colour	Brooder Compartments Opened (days)	Aisle Width (m)	Aisle Length (m)	Total Chicks Placed	Average Group Size	Natural Light	Migration Fences	Visits When Ramps Available
1A	1	Lohmann	Brown	46	1.6	102.4	29 600	14 800	no	yes	2, 3
1B	1	Dekalb	White	48	1.5	122.8	32 742	10 914	no	yes	2,3
1C[1]	1	Lohmann	Brown	33	1.9	104.0	47 000	2 500	no	yes	2
1D[1]	1	Lohmann	White	33	1.9	104.0	47 000	3 000	no	yes	2
1E[1]	1	Dekalb	White	28	2.3	104.0	47 000	5 167	no	yes	2
2A	2	Lohmann	White	28	1.4	50.1	43 000	5 591	no	no	2, 3
2B	2	Bovan	Brown	28	1.7	64.5	35 506	6 642	no	no	2, 3
2C[2]	2	Lohmann	White	28	21.3	82.7	39 200	7 350	yes	no	2, 3
2D	2	Shaver	White	22	1.4	33.6	11 200	4 200	no	no	none
2E[3]	2	Lohmann	Brown	28	2.0	57.9	22 470	4 214	yes	no	2,3
3A	3	Lohmann	White	42	2.7	48.0	14 240	14 240	no	no	1,2
3B[3]	3	Lohmann	Brown	49	2.0	67.8	20 700	20 700	yes	no	2
3C[4]	3	Lohmann	Brown	91	2.7	42.7	9 200	9 200	no	no	2
3D	3	ISA	Brown	45	3.3	7.2	700	700	no	no	2,3
3E	3	Lohmann	White	46	1.8	34.0	11 730	11 730	no	no	1,2,3

[1]Flocks 1C, 1D, and 1E were simultaneously housed same building and separated by migration fences. There were 10 000 Lohmann Brown, 6 000 Lohmann White, and 31 000 Dekalb White chicks placed.

[2]Non-organic flock provided natural light

[3]Organic flocks provided natural light

[4]Organic flock not provided natural light
