# Supplementary material for: Effects of Rearing Aviary Style and Genetic Strain on the Locomotion and Musculoskeletal Characteristics of Layer Pullets
Source: Animals (Basel). 2021 Feb 27;11(3):634. doi: 10.3390/ani11030634 (PMC7997280; doi:10.3390/ani11030634)
Supplement: Supplementary file 1 [file animals-11-00634-s001.zip › APufall_SupplementaryMaterial_S2.pdf]

**Table S2.** Ages at which measures were taken and hours of light provided across all visits

| | **Visit 1** | | | **Visit 2** | | | | **Visit 3** | | | |
|---|---|---|---|---|---|---|---|---|---|---|---|
| **Flock** | Age weights taken (days) | Age videos taken (days) | Hours Light | Age weights taken (days) | Age videos taken (days) | Hours Light | Age Birds euthanized (days) | Age weights taken (days) | Age videos taken (days) | Hours Light | Age Birds euthanized (days) |
| 1A | 40 | 41 | 10 | 73 | 74 | 9 | 75 | 119 | 120 | 10 | 119 |
| 1B | 39 | 40 | 13 | 65 | 66 | 11 | 65 | 106 | 107 | 11 | 106 |
| 1C | 20 | 21 | 19 | 61 | 62 | 9 | . | 105 | 112 | 10 | . |
| 1D | 20 | 21 | 19 | 61 | 62 | 9 | . | 105 | 112 | 10 | . |
| 1E | 16 | 17 | 19 | 56 | 57 | 9 | 56 | 97 | 108 | 10 | . |
| 2A | 23 | 24 | 12 | 72 | 73 | 10 | 73 | 115 | 116 | 10.5 | 115 |
| 2B | 21 | 22 | 8 | 66 | 67 | 8 | 67 | 112 | 113 | 10 | 112 |
| 2C | 19 | 20 | 16 | 70 | 71 | 9 | 70 | 112 | 113 | 9 | 112 |
| 2D | 23 | 24 | 14 | 69 | 70 | 10 | 69 | 111 | 112 | 10 | 111 |
| 2E | 25 | 26 | 13 | 70 | 71 | 9 | 70 | 112 | 113 | 9.5 | 113 |
| 3A | 25 | 26 | 16 | 62 | 63 | 11 | 63 | 109 | 109 | 11 | 108 |
| 3B | 25 | 26 | 14 | 67 | 68 | 9 | 68 | 113 | 114 | 9 | 113 |
| 3C | 27 | 28 | 15 | 70 | 71 | 10 | 71 | 108 | 109 | 10 | 108 |
| 3D | 23 | 24 | 8 | 70 | 71 | 8 | 71 | 111 | 112 | 8 | 111 |
| 3E | 28 | 29 | 14 | 71 | 72 | 11 | 72 | 109 | 110 | 11 | 110 |
